# Computed tomography-based radiomics machine learning models for prediction of histological invasiveness with sub-centimeter subsolid pulmonary nodules: a retrospective study

**DOI:** 10.7717/peerj.14559

**Published:** 2023-01-10

**Authors:** Haochuan Zhang, Shixiong Wang, Zhenkai Deng, Yangli Li, Yingying Yang, He Huang

**Affiliations:** Department of Pulmonary and Critical Care Medicine, The First Affiliated Hospital of Shenzhen University, Shenzhen Second People’s Hospital, Graduate School of Guangzhou Medical University, Shenzhen, China

**Keywords:** Radiomics, Machine learning, CT images, Sub-centimeter subsolid pulmonary nodules, Invasiveness

## Abstract

To improve the accuracy of preoperative diagnoses and avoid over- or undertreatment, we aimed to develop and compare computed tomography-based radiomics machine learning models for the prediction of histological invasiveness using sub-centimeter subsolid pulmonary nodules. Three predictive models based on radiomics were built using three machine learning classifiers to discriminate the invasiveness of the sub-centimeter subsolid pulmonary nodules. A total of 203 sub-centimeter nodules from 177 patients were collected and assigned randomly to the training set (*n* = 143) or test set (*n* = 60). The areas under the curve of the predictive models were 0.743 (95% confidence interval CI [0.661–0.824]) for the logistic regression, 0.828 (95% CI [0.76–0.896]) for the support vector machine, and 0.917 (95% CI [0.869–0.965]) for the XGBoost classifier models in the training set, and 0.803 (95% CI [0.694–0.913]), 0.726 (95% CI [0.598–0.854]), and 0.874 (95% CI [0.776–0.972]) in the test set, respectively. In addition, the decision curve showed that the XGBoost model added more net benefit within the range of 0.06 to 0.93.

## Introduction

Owing to advances in medical imaging technology and the wide application of low-dose computed tomography (CT), the detection of pulmonary nodules has drastically increased over the past decade. Among these, subsolid nodules (SSNs), which behave as ground-glass opacities on a CT scan, have drawn significant attention. The Dutch–Belgian Randomized Lung Cancer Screening Trial (Nederlands Leuvens Longkanker Screenings Onderzoek; NELSON) has shown that SSNs are detected in 3.3% of patients (Scholten et al., 2015). In another study in Chinese medical staff, SSNs diagnosed as adenocarcinoma via histopathology were detected in 2.0% of cases (Zhang et al., 2020). However, preoperative diagnose of SSNs remains an unresolved issue, and the accurate diagnosis and detection of early triggers have become a key topic in public health. In the present study, we focused specifically on the subtype of sub-centimeter SSNs.

The pulmonary nodule is defined as an area of increased attenuation of the lung in a CT scan with a diameter of less than 30 mm (Erasmus et al., 2000). Depending on the component of the nodule, it can be classified into two subtypes: a solid or a subsolid nodule. A subsolid nodule is defined as an area that partly or entirely disappears in the mediastinal window of a CT scan (Bueno, Landeras & Chung, 2018; Clark et al., 2015) and presents with a variety of histopathological findings. On the one hand, it can be a benign lesion, such as focal fibrosis, inflammation, bleeding, or a precancerous lesion (*e.g.*, atypical adenomatous hyperplasia [AAH]). On the other hand, it can be adenocarcinoma *in situ* (AIS), minimally invasive adenocarcinoma (MIA), or invasive adenocarcinoma (IAC) (Henschke et al., 2002; Hutchinson, Moreira & Ko, 2017; Travis et al., 2015). Usually, AAH and AIS are considered preinvasive lesions, whereas MIA and IAC are regarded as invasive lesions (Travis et al., 2015).

The screening of I-ELCAP (the International Early Lung Cancer Action Program) indicated that 34% of SSNs are malignant, whereas only 7% of solid nodules are malignant (Yip et al., 2015). According to the international multidisciplinary classification of lung adenocarcinoma published in 2015, wedge resection and segmental resection without lymph node dissection are recommended before lesions reach the level of invasive adenocarcinoma. Conversely, for IAC, lobectomy and lymph node dissection are recommended (Goldstraw et al., 2016; Liu et al., 2016). Moreover, Fu et al. (2021a) reported that SSNs with a diameter of more than 10 mm are more often diagnosed as IAC. Therefore, preoperative diagnosis of sub-centimeter SSNs is crucial. To date, numerous studies have focused on SSNs; however, research on sub-centimeter nodules is scarce (Meng et al., 2021; She et al., 2017; Sun et al., 2019; Sun et al., 2020). Therefore, improving the accuracy of preoperative diagnoses of subsolid sub-centimeter nodules and providing a reference for clinicians is particularly important.

At present, preoperative diagnosis methods for sub-centimeter SSNs include (Godoy & Naidich, 2012), (1) CT characteristics, such as diameter, density, spiculation, lobulation, cavity, bubble-like lucency, air bronchogram, vascular breakthrough sign, and pleural indentation sign; (2) positron emission tomography (PET)-CT; and (3) biopsy. The limitation of CT characteristics is the difficulty in achieving interobserver agreement of findings. The limitation of PET-CT is that the maximum standard uptake values of SSNs are generally low (Nomori et al., 2004). Successful biopsy cannot be guaranteed because the diameter of the nodule is small, and the rate of false negatives is high (Ng et al., 2008; Ricciardi et al., 2021). Moreover, biopsy is a highly invasive method. Totally, there is an urgent need for a different approach to provide reference for preoperative decisions.

In 2012, Lambin et al. (2017) developed the concept of radiomics, which overcame the limitations of existing techniques (Lambin et al., 2012). Currently, radiomics has been applied widely to diagnosis, staging, efficacy assessment, and prognosis. Moreover, a considerable number of studies have demonstrated that radiomics is helpful for the preoperative diagnosis of SSNs (Meng et al., 2021; She et al., 2017; Sun et al., 2019; Sun et al., 2020). Therefore, we conducted a retrospective review of patients who had undergone surgery for sub-centimeter SSNs to evaluate the performance of CT-based radiomics machine learning models for discriminating the invasiveness of subsolid sub-centimeter nodules.

## Materials and Methods

### Patient selection

In this retrospective study, we obtained the medical records of patients who had undergone surgical resection at our hospital from 2019 to 2021 and whose nodules had been confirmed to be adenocarcinoma spectrum lesions. The cases met the following inclusion criteria: (1) the maximum diameter of the SSN in the largest slice in the CT scan was ≤ 10 mm; (2) the lesion was confirmed as AAH, AIS, MIA, or IAC via histopathology; (3) the preoperative CT scan had a slice thickness of ≤ 1.5 mm; (4) the interval between the preoperative CT scan and surgical resection was ≤ 1 month. The exclusion criteria for the study were: (1) confirmation of histopathological result by biopsy or bronchoscopy; (2) the patient had undergone other therapies for tumors, such as chemotherapy, radiotherapy, and targeted therapy. The cases were divided into two groups depending on histological invasiveness: Group A (AAH/AIS/MIA) and Group B (IAC). According to the ratio of cases in Group A and B, the cases were divided into two sets: 70% of cases were divided into the training set, and 30% of cases were divided into the test set.

### CT image acquisition

The CT examination was performed on a Definition AS 40-detector (Siemens Healthineers, Erlangen, Germany), a Definition Flash 64-detector and Dual-energy Force (Siemens Healthineers), a Somatom EMOTION 16-detector (Siemens Healthineers), and an IQon 64-detector and Dual-energy Force (Philips Healthcare, Amsterdam, Netherlands). Scans covered the area between the thorax inlet and the bilateral adrenal glands in the supine position. The scanning parameters were as follows: 120 kV, automatic tube current, pitch 0.75–1.5, field of view 336 × 336 mm, and resolution 512 × 512. CT images were interpreted by two experienced pulmonologists who were blinded to the histopathological diagnosis in the lung and mediastinal windows. The following subjective features of the lesion were recorded: (1) component of the lesion (part solid or non-solid), (2) lesion diameter (the maximum diameter in the largest slice), (3) lesion location, and (4) the characteristics of the lesion, including lobulation (absent, present), spiculation (absent, present), bubble-like lucency (absent, present), cavity (absent, present), pleural indentation sign (absent, present), vascular breakthrough sign (absent, present), sharp (round or oval, irregular or polygonal), and margin (clear, unclear).

### Histopathological analysis

Surgical specimens were analyzed separately by two experienced pathologists blinded to the CT findings, according to the 2015 International Association for the Study of Lung Cancer/American Thoracic Society, the European Respiratory Society classification system 2015 (McKee et al., 2016), and the World Health Organization classification of lung neoplasms (Travis et al., 2011).

### Nodule segmentation

**Figure 1:**
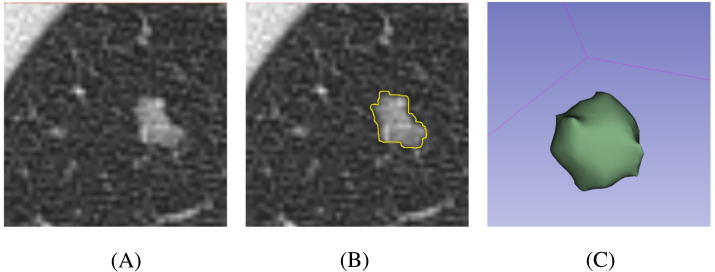
Schematic processes of the nodule segmentation. (A) Original CT image of the nodule; (B) 2D segmentation result of the nodule; (C) 3D volumetric reconstruction of the nodule.

At present, semi-automatic segmentation remains a relatively reliable method (Owens et al., 2018). Segmentation was performed using 3D Slicer 4.11 (https://slicer.org/). First, CT images were downloaded to the workstation from the picture archiving and communication system. Second, one pulmonologist outlined the margin of the nodule slice-by-slice. The software then automatically calculated the region of interest (ROI) in three dimensions according to the selected region (Fig. 1). In addition, the mean and standard deviation (SD) of the CT attenuation of the lesion were calculated by the software. Finally, another pulmonologist reviewed the processes and results. In order to reduce the heterogeneity bias caused by different CT scanners or parameters, ROIs were resampled after segmentation.

### Radiomics feature extraction

Radiomics feature extraction was performed using pyRadiomics (https://pyradiomics.readthedocs.io/), which is an open-source Python package. The types of features extracted included: (1) first-order statistics; (2) shape-based 3D; (3) shape-based 2D; (4) Gray Level Co-occurrence Matrix (GLCM); (5) Gray Level Run Length Matrix (GLRLM); (6) Gray Level Size Zone Matrix (GLSZM); (7) Neighboring Gray Tone Difference Matrix (NGTDM); and (8) Gray Level Dependence Matrix (GLDM). In addition, there were eight filters that could transform the original images to obtain further features from the transformed images, which included: (1) Wavelet; (2) LoG; (3) Square; (4) SquareRoot; (5) Logarithm; (6) Exponential; (7) Gradient; (8) Local Binary Pattern 2D (LBP2D); and (9) Local Binary Pattern 3D (LBP3D). In total, more than 1,700 radiomics features were extracted.

### Radiomics feature selection and predict model construction

The process of radiomics feature selection from the radiomics feature data extracted from the training set was as follows: (1) removal of the redundant features with a Pearson correlation coefficient >0.9; (2) removal of the features with a *p* > 0.05 in the Mann–Whitney U test; (3) retaining features filtered by the least absolute shrinkage and selection operator (LASSO). Using the retained features, three machine learning models were constructed according to three classifiers using the training set for differentiating Group A (AAH/AIS/MIA) from Group B (IAC). The classifiers included a logistic regression (LR) classifier, a support vector machine (SVM) classifier, and an XGBoost (XGB) classifier. After the models were constructed, the training dataset and test dataset were fitted to the model to calculate the area under the receiver operator characteristic (ROC) curve (AUC), sensitivity, specificity, and accuracy. Differences in the ROC curves between the training and test sets were assessed using the Delong test. To compare the clinical benefit between the different models, we performed decision curve analysis (DCA) by calculating the net benefit for a range of threshold probabilities based on all datasets (Vickers et al., 2008).

### Statistical analysis

All statistical analyses and calculations were conducted using R version 4.2.0 (https://www.r-project.org/) (R Core Team, 2022). LASSO was performed using “glmnet”. The ROC analysis and DeLong tests were performed using “pROC”. DCA was plotted using “rmda”. Data division and model construction were conducted using “caret”, “kernlab”, and “XGBoost”. AUCs are presented with 95% confidence intervals (CIs), which were bootstrap bias corrected. All statistical tests were two-tailed, and *p*-values less than 0.05 were considered statistically significant. The differences of continuous variables were assessed by Kruskal-Wallis Rank Sum test. The differences of categorical variables were assessed by Fisher’s exact test.

### Ethics approval and consent to participate

Our study was approved by Shenzhen Second People’s Hospital Clinical Research Ethics Committee, and the requirement for informed consent was waived. (Approval Number: 2022035).

## Results

The characteristics of the cases are summarized in Table 1. A total of 203 sub-centimeter nodules from 177 patients (49 males, 128 females; mean age: 50.6 ± 12.04 years) were included and assigned randomly to the training set (*n* = 143) or test set (*n* = 60). Among the lesions, 104 SSNs were confirmed as IAC and 99 were classified as AAH/AIS/MIA. No significant differences in age, sex, component, location, lobulation, spiculation, bubble-like lucency, cavity, pleural indentation sign, sharp, or margin were found between Group A (AAH/AIS/IAC) and B (IAC) in either the training or test sets. However, the mean and SD of CT attenuation differed significantly between the two groups (*p* < 0.05).

**Table 1:** Summary of the characteristics of cases.

	Training Set				Test Set			
	Total (*n* = 143)	AAH/AIS/MIA (*n* = 70)	IAC (*n* = 73)	*p*	Total (*n* = 60)	AAH/AIS/MIA (*n* = 29)	IAC (*n* = 31)	*p*
AGE (years)	50.0 ± 11.4	49.1 ± 10.0	50.8 ± 12.5	0.356	51.9 ± 13.5	50.1 ± 13.2	53.5 ± 13.8	0.337
SEX				0.149				0.438
Male	43 (30.1%)	25 (35.7%)	18 (24.7%)		12 (20.0%)	7 (24.1%)	5 (16.1%)	
Female	100 (69.9%)	45 (64.3%)	55 (75.3%)		48 (80.0%)	22 (75.9%)	26 (83.9%)	
DIAMETER (mm)	0.73 ± 0.19	0.69 ± 0.20	0.77 ± 0.18	0.025	0.73 ± 0.20	0.68 ± 0.17	0.77 ± 0.21	0.061
COMPONENT				0.258				0.881
Part-solid	110 (76.9%)	51 (72.9%)	59 (80.8%)		45 (75.0%)	22 (75.9%)	23 (74.2%)	
Non-solid	33 (23.1%)	19 (27.1%)	14 (19.2%)		15 (25.0%)	7 (24.1%)	8 (25.8%)	
LOCATION				0.674				0.695
Left Upper Lobe	43 (30.1%)	25 (35.7%)	18 (24.7%)		19 (31.7%)	11 (37.9%)	8 (25.8%)	
Left Lower Lobe	15 (10.5%)	7 (10.0%)	8 (11.0%)		13 (21.7%)	6 (20.7%)	7 (22.6%)	
Right Upper Lobe	38 (26.6%)	16 (22.9%)	22 (30.1%)		16 (26.7%)	7 (24.1%)	9 (29.0%)	
Right Middle Lobe	10 (7.0%)	5 (7.1%)	5 (6.8%)		7 (11.7%)	2 (6.9%)	5 (16.1%)	
Right Lower Lobe	37 (25.9%)	17 (24.3%)	20 (27.4%)		5 (8.3%)	3 (10.3%)	2 (6.5%)	
LOBULATION				0.711				0.931
Absent	130 (90.9%)	63 (90.0%)	67 (91.8%)		54 (90.0%)	26 (89.7%)	28 (90.3%)	
Present	13 (9.1%)	7 (10.0%)	6 (8.2%)		6 (10.0%)	3 (10.3%)	3 (9.7%)	
SPICULATION				0.324				0.638
Absent	118 (82.5%)	60 (85.7%)	58 (79.5%)		51 (85.0%)	24 (82.8%)	27 (87.1%)	
Present	25 (17.5%)	10 (14.3%)	15 (20.5%)		9 (15.0%)	5 (17.2%)	4 (12.9%)	
BUBBLE-LIKE LUCENCY				0.581				0.460
Absent	83 (58.0%)	39 (55.7%)	44 (60.3%)		36 (60.0%)	16 (55.2%)	20 (64.5%)	
Present	60 (42.0%)	31 (44.3%)	29 (39.7%)		24 (40.0%)	13 (44.8%)	11 (35.5%)	
CAVITY				0.780				0.962
Absent	134 (93.7%)	66 (94.3%)	68 (93.2%)		58 (96.7%)	28 (96.6%)	30 (96.8%)	
Present	9 (6.3%)	4 (5.7%)	5 (6.8%)		2 (3.3%)	1 (3.4%)	1 (3.2%)	
PLERUAL INDENTATION SIGN				0.780				0.185
Absent	134 (93.7%)	66 (94.3%)	68 (93.2%)		55 (91.7%)	28 (96.6%)	27 (87.1%)	
Present	9 (6.3%)	4 (5.7%)	5 (6.8%)		5 (8.3%)	1 (3.4%)	4 (12.9%)	
VASCULAR BREAKTHROU-GH SIGN				0.004				0.655
Absent	47 (32.9%)	31 (44.3%)	16 (21.9%)		15 (25.0%)	8 (27.6%)	7 (22.6%)	
Present	96 (67.1%)	39 (55.7%)	57 (78.1%)		45 (75.0%)	21 (72.4%)	24 (77.4%)	
SHARP				0.832				0.931
Round or oval	130 (90.9%)	64 (91.4%)	66 (90.4%)		54 (90.0%)	26 (89.7%)	28 (90.3%)	
Irregular or polygonal	13 (9.1%)	6 (8.6%)	7 (9.6%)		6 (10.0%)	3 (10.3%)	3 (9.7%)	
MARGIN				0.080				0.833
Clear	90 (62.9%)	39 (55.7%)	51 (69.9%)		36 (60.0%)	17 (58.6%)	19 (61.3%)	
Unclear	53 (37.1%)	31 (44.3%)	22 (30.1%)		24 (40.0%)	12 (41.4%)	12 (38.7%)	
Mean CT attenuation (HU)	−663.26 ± 102.56	−685.36 ± 93.10	−642.06 ± 107.28	0.011	−657.46 ± 99.14	−687.61 ± 76.30	−629.26 ± 110.44	0.021
SD of CT attenuation (HU)	141.1 ± 54.9	129.8 ± 55.1	151.9 ± 52.7	0.015	145.3 ± 62.4	127.0 ± 55.0	162.5 ± 64.9	0.026

**Notes.**

The differences of continuous variables were assessed with the Kruskal-Wallis Rank Sum test. The differences of categorical variables were assessed by Fisher’s exact test.

**Figure 2:**
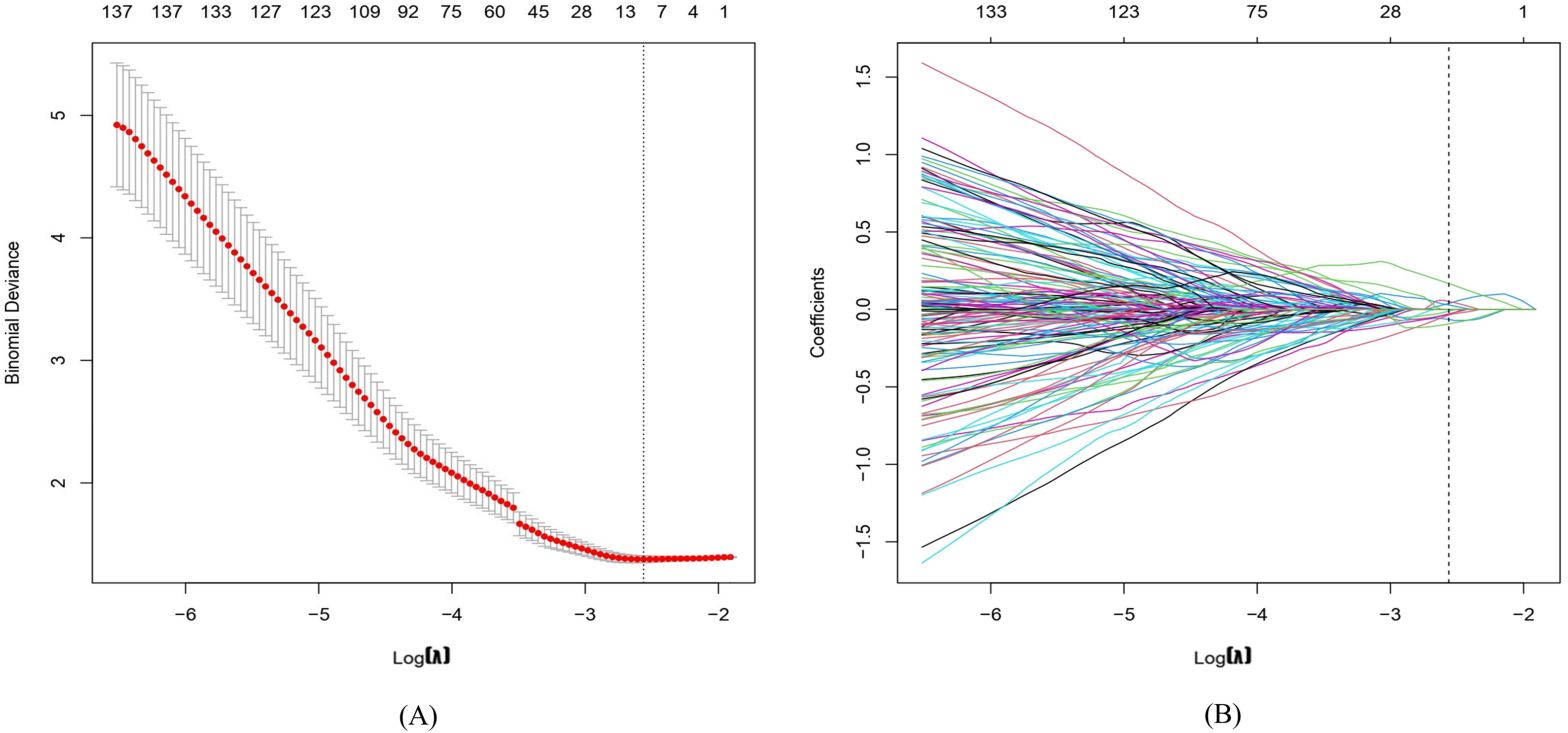
Radiomics features are selected via least absolute shrinkage and selection operator (LASSO) logistic regression. (A) The tuning parameter (*λ*) is selected by 10-fold cross-validation with minimum criteria. Log(*λ*) (*x*-axis) was used to plot the binomial deviance (*y*-axis). The dotted vertical lines were drawn at the optimal value of *λ*, which is the point where the model fits the data best. Log(*λ*) = −2.562, while *λ* was optimally valued at 0.07718. (B) The LASSO coefficient profiles of all the features. As shown in the plot, the dotted vertical line corresponds to the value selected by k-fold (*k* = 10) cross-validation, where 10 optimal features with non-zero coefficients were indicated in the plot.

**Figure 3:**
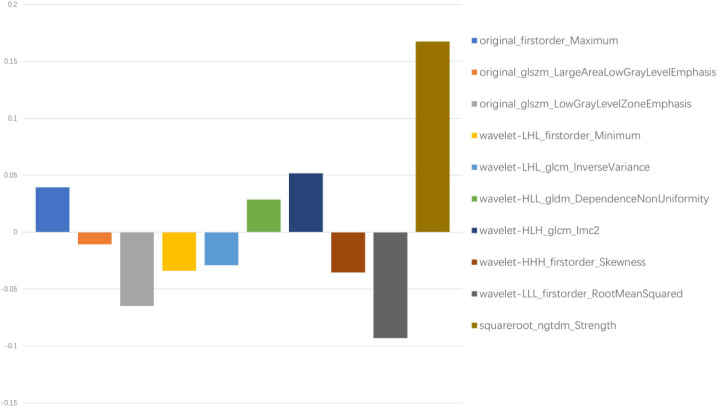
Ten filtered radiomics features with non-zero coefficients. *Y*-axis represented the values of coefficients.

In total, 1,781 radiomics features were extracted. After correlation analysis and the Mann–Whitney U tests, 1027 radiomics features were retained. The LASSO with a k-fold (*k* = 10) cross-validation yielded an optimal *λ* value of 0.07718 and a corresponding log (*λ*) value of −2.562. There were 10 radiomics features with non-zero coefficients (Figs. 2 and 3), which included firstorder_Maximum, glszm_LargeAreaLowGrayLevelEmphasis, glszm_LowGrayLevelZoneEmphasis, wavelet-LHL_firstorder_Minimum, wavelet-LHL_glcm_InverseVariance, wavelet-HLL_gldm_DependenceNonUniformity, wavelet-HLH_glcm_Imc2, wavelet-HHH_firstorder_Skewness, wavelet-LLL_firstorder_RootMeanSquared, and squareroot_ngtdm_Strength.

**Figure 4:**
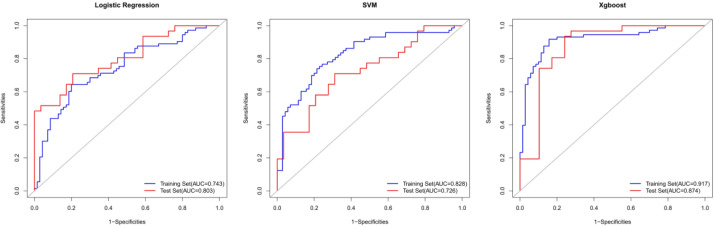
The ROC curves for the diagnosis of three radiomics models in the training set and test sets.

**Figure 5:**
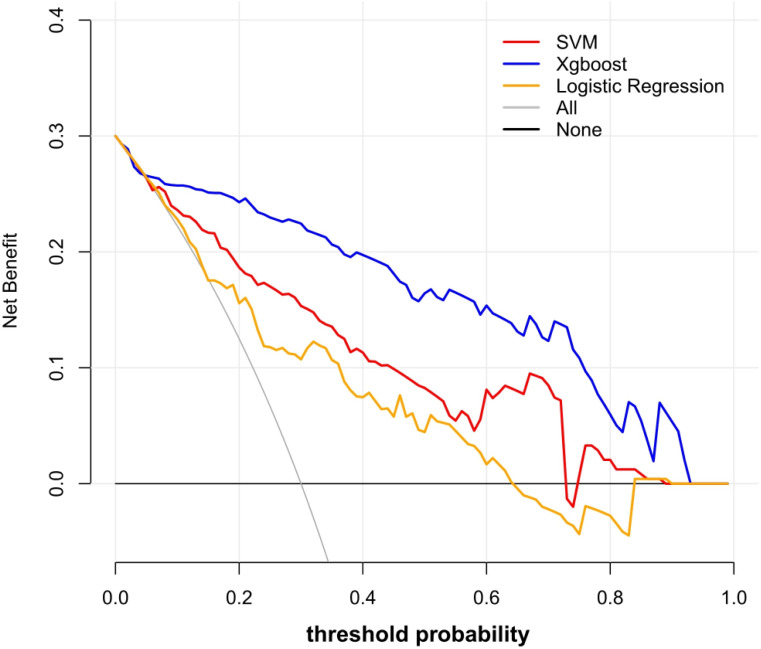
Decision curve analysis of the prediction models. The *y*-axis represented the net benefit. The red line represented the SVM model. The blue line represents the XGB model. The yellow line represented the LR model. The gray line represented the assumption that all patients had ICA. The black line represented the hypothesis that none of patients had ICA. The *x*-axis represented the threshold probability. The threshold probability was where the expected benefit of treatment as ICA was equal to the expected benefit of treatment as AAH/AIS/MIA. The decision curve showed that the XGB model added more net benefit than the SVM model or the LR model within the range of 0.06 to 0.93.

Using these 10 radiomics features, three machine learning models were built using the LR, SVM, and XGB classifiers. An overview of the results is provided in Table 2 and Fig. 4. In the training set, the predictive accuracies of the three models were 0.720 for LR, 0.762 for SVM, and 0.881 for XGB. Those in the test set were 0.750 for LR, 0.700 for SVM, and 0.850 for XGB. The AUCs of the predictive models were 0.743 (95% CI [0.661–0.824]) for LR, 0.828 (95% CI [0.760–0.896]) for SVM, and 0.917 (95% CI [0.869–0.965]) for XGB in the training set, and 0.803 (95% CI [0.694–0.913]) for LR, 0.726 (95% CI [0.598–0.854]) for SVM, and 0.874 (95% CI [0.776–0.972]) for XGB in the test set. The predictive performance of the radiomics models in the test set was not significantly different from that in the training set (Delong tests: *p* > 0.05). These results indicated that the machine learning models were beneficial for discriminating between IAC and AAH/AIS/MIA with subsolid sub-centimeter pulmonary nodules. The DCA results of the three models are presented in Fig. 5. The decision curve showed that the XGB model offered a greater net benefit than the LR and SVM models within the threshold probability ranges of 0.06 to 0.93. Among the three predictive models, the XGB model had the best performance.

## Discussion

For many years, SSNs were considered one of the imaging manifestations of pneumonia until it was revealed that SSNs can be early-stage lung cancer (Jang et al., 1996; Kuriyama et al., 1999). Because lung cancer is usually asymptomatic in the early stage, early detection is challenging. The global lung cancer mortality rate is the highest of all cancers, with 1.08 million deaths in 2020 (Ferlay et al., 2020). Moreover, there has been a significant increase in the prevalence of adenocarcinoma, which is now the most prevalent type of lung cancer, accounting for 50% of all lung cancer diagnoses (Bray et al., 2018; Nagy-Mignotte et al., 2011; Barta, Powell & Wisnivesky, 2019). Thus, lung adenocarcinoma is a highly relevant topic in public health research.

**Table 2:** Performance of the three radiomics models on the training sets and the test sets.

	Training Set				Test Set				Delong Test
	AUC (95%CI)	Accuracy	Sensitivity	Specialty	AUC (95%CI)	Accuracy	Sensitivity	Specialty	*p*
LR	0.743 (0.661-0.824)	0.720	0.644	0.800	0.803 (0.694-0.913)	0.750	0.710	0.793	0.387
SVM	0.828 (0.76-0.896)	0.762	0.740	0.786	0.726 (0.598-0.854)	0.700	0.710	0.690	0.172
XGB	0.917 (0.869-0.965)	0.881	0.918	0.843	0.874 (0.776-0.972)	0.850	0.935	0.759	0.446

In CT images, lung adenocarcinomas may appear as ground-glass nodules during the earliest stages. In histopathology, AAH, AIS, MIA, and IAC are considered a spectrum of adenocarcinomas (Bray et al., 2018). In the lining of alveolar walls or respiratory bronchioles, AAH is characterized by a limited proliferation of mildly to moderately atypical type II pneumocytes and/or Clara cells. AIS is a localized small adenocarcinoma that grows within pre-existing alveolar structures, without invaders from the stroma, blood vessels, or pleura. MIA is a small, solitary adenocarcinoma, with a predominantly lepidic pattern and a maximal 5-mm invasion in the greatest focus. The prognosis of IAC differs significantly from that of MIA and AIS. Following surgery for a preinvasive lesion and MIA, patients have a 5-year survival rate of roughly 100%. In contrast, the progression-free survival rate for patients with IAC is 74.6% (Goldstraw et al., 2016; Liu et al., 2016). Thus, a well-performing predictive model using radiomics features and machine learning may provide a reference for surgeons and the public.

Zhang et al. (2019) constructed a histogram analysis and reported a sensitivity of 0.794 and specificity of 0.914 for differentiating AIS/MIA from IAC. However, their inclusion criteria included lesions measuring ≤ 30 mm. Shi et al. (2021) demonstrated that radiomic features could be applied for predicting the invasiveness of SSNs measuring 5–10 mm in diameter; the AUC of their model was only 0.831 in the training set and 0.792 in an internal test set. Jiang et al. (2021) also used radiomics features to distinguish IAC from MIA (accuracy = 0.768, AUC = 0.892), but their study only included 53 cases of IAC and 47 cases of MIA. Our study developed novel models for predicting the risk of IAC from sub-centimeter subsolid nodule imaging data with good performance. In particular, the XGB model showed an accuracy of 0.881 in the training set and 0.850 in the test set for differentiating IAC from AAH/AIS/MIA. The sensitivity and specificity were all above 0.750 in both sets. In addition, ROC curves revealed AUCs of 0.917 (95% CI [0.869–0.965]) and 0.874 (95% CI [0.776–0.972]) for the training and test sets, respectively, demonstrating that the radiomics machine learning models may provide a reference for surgeons to evaluate the invasiveness of sub-centimeter SSNs accurately and avoid over- or undertreatment. In addition, DCA demonstrated that the XGBoost model was more clinically useful.

Usually, objective CT characteristics are used to evaluate the invasiveness of SSNs in clinical practice (Ricciardi et al., 2021). However, it’s not reliable when we evaluate the sub-centimeter SSNs. Firstly, the effectiveness of using objective CT characteristics is still controversial. Some studies suggested the mean and SD of CT attenuation as factors for evaluating the invasiveness of SSNs (She et al., 2017; Kitami et al., 2016), but other studies have reported no correlation between mean and SD of CT attenuation and invasiveness and shown that only lesion size is the influencing factor (Fu et al., 2021b). Whether the mean and SD of CT attenuation reflect the invasiveness of SSNs remains inconclusive. Secondly, in contrast to larger nodules, sub-centimeter SSNs lack obvious different morphological CT signs, such as lobulation, spiculation, cavity, and bubble-like lucency (Chen et al., 2021; Wu et al., 2017). Similarly, the present analysis showed that these morphological CT signs were not independent predictors of IAC, indicating that nodules without these morphological features should not be ignored. Thirdly, agreement on evaluations of morphological CT signs between clinicians is often low (Zhao et al., 2019).

In contrast to objective CT features, radiomics converts image data into quantitative data using high-throughput mining. The massive data obtained contribute to diversity analysis in higher dimensions (Lambin et al., 2012; Lambin et al., 2017). Radiomics features are considered to quantitatively reflect the heterogeneity of lesions, which can predict tumor behavior (Lambin et al., 2012; Lambin et al., 2017; Nioche et al., 2018; Chen et al., 2020). Previous large-scale studies have confirmed that radiomics features exhibit good diagnostic performance. Chen et al. (2018) demonstrated 76 relevant radiomics features selected from 750 extracted features that differed significantly between benign and malignant pulmonary nodules. Hawkins et al. (2016) reported that radiomics could be applied to screen the risk of lung cancer (accuracy = 0.800). In the present study, 1,781 radiomics features were extracted from the ROIs of lesions selected using multiple methods. At last, we identified 10 radiomics features that were more strongly associated with invasiveness than other features. The results demonstrated that the radiomics models reached satisfactory prediction performance, and the Delong tests between the training and test sets showed stability of the radiomics models, which may help clinicians in selecting appropriate treatment measures.

Further, our models were constructed using three different machine learning classifiers. Machine learning has been applied widely and validated to improve predictive performance. Previous studies have commonly used LR, random forest, and SVM as classifiers to build predictive models (Huang et al., 2016; Parmar et al., 2015; Yang et al., 2015; Ypsilantis et al., 2015). However, in the present study, we selected a relatively novel classifier, XGB, and compared it with the other two classifiers, revealing that XGB model had the best performance when assessed by ROC curve analysis and DCA. The XGB model may provide a better strategy for diagnosis in clinical practice. In fact, the XGB classifier has been demonstrated to have high flexibility in other fields (Dhaliwal, Nahid & Abbas, 2018; Gumus & Kiran, 2017; Ren et al., 2017; Torlay et al., 2017). In the Kaggle data science competition, more than half of the challenge-winning solutions used XGB models (Chen & Guestrin, 2016). Regularization of L1 and L2 in XGB prevents the model from overfitting and takes advantage of parallel processing (Tang et al., 2019), which likely contributes to its superiority.

We also acknowledge that our research has limitations. First of all, the present study design was retrospective and the models were not validated using an external dataset. Therefore, a multicenter clinical trial with a larger sample size and prospective validation set are needed to determine the generalization of the models. Besides, our data showed that the mean and SD of CT attenuation helped to distinguish the invasiveness of lesions and the vascular breakthrough sign with in the training set. Our next plan is to identify more meaningful clinical features to construct a combined model to enhance predictive performance. Moreover, because only surgically cases were included, the cases inevitably were skewed toward the malignant nodules in morphology. Thus, more cases are supposed to be allowed for the inclusion of study.

## Conclusion

Our newly developed prediction models were able to distinguish between IAC and AAH/AIS/MIA. We revealed that radiomics features reflect the heterogeneity and invasiveness of sub-centimeter SSNs. These models may offer a noninvasive and convenient method to improve preoperative prediction for surgeons to avoid over- or undertreatment. The predictive performance of the models will be further improved by including additional clinical factors and validating using multicenter datasets.

##  Supplemental Information

10.7717/peerj.14559/supp-1 Supplemental Information 1 The clinical characteristics of cases

10.7717/peerj.14559/supp-2 Supplemental Information 2 The data of radiomics features extracted from cases

10.7717/peerj.14559/supp-3 Supplemental Information 3 Codebook for the raw data

## Abbreviations

CT: Computed tomography
AUC: Operating characteristic curve
LR: Logistics regression
SVM: Support vector machine
XGB: XGBoost
SSNs: Subsolid nodules
AAH: Atypical adenomatous hyperplasia
AIS: Adenocarcinoma *in situ*
MIA: Minimally invasive adenocarcinoma
IAC: Invasive adenocarcinoma
SUV: Standard uptake value
WHO: World Health Organization
LASSO: Least absolute shrinkage and selection operator
ROI: Region of interest
ROC: Receiver operating characteristic
DCA: Decision curve analysis
CI: Confidence interval

## Additional Information and Declarations

### Data Availability

The following information was supplied regarding data availability: The raw measurements are available in the Supplemental Files.

## References

[ref-1] Barta JA, Powell CA, Wisnivesky JP (2019). Global epidemiology of lung cancer. Annals of Global Health.

[ref-2] Bray F, Ferlay J, Soerjomataram I, Siegel RL, Torre LA, Jemal A (2018). Global cancer statistics 2018: GLOBOCAN estimates of incidence and mortality worldwide for 36 cancers in 185 countries. CA: A Cancer Journal for Clinicians.

[ref-3] Bueno J, Landeras L, Chung JH (2018). Updated fleischner society guidelines for managing incidental pulmonary nodules: common questions and challenging scenarios. Radiographics.

[ref-4] Chen CH, Chang CK, Tu CY, Liao WC, Wu BR, Chou KT, Chiou YR, Yang SN, Zhang G, Huang TC (2018). Radiomic features analysis in computed tomography images of lung nodule classification. PLOS ONE.

[ref-5] Chen T, Guestrin C (2016). XGBoost: a scalable tree boosting system.

[ref-6] Chen W, Li M, Mao D, Ge X, Wang J, Tan M, Ma W, Huang X, Lu J, Li C, Hua Y, Wu H (2021). Radiomics signature on CECT as a predictive factor for invasiveness of lung adenocarcinoma manifesting as subcentimeter ground glass nodules. Scientific Reports.

[ref-7] Chen X, Feng B, Chen Y, Liu K, Li K, Duan X, Hao Y, Cui E, Liu Z, Zhang C, Long W, Liu X (2020). A CT-based radiomics nomogram for prediction of lung adenocarcinomas and granulomatous lesions in patient with solitary sub-centimeter solid nodules. Cancer Imaging.

[ref-8] Clark TJ, Flood TF, Maximin ST, Sachs PB (2015). Lung CT screening reporting and data system speed and accuracy are increased with the use of a semiautomated computer application. Journal of the American College of Radiology.

[ref-9] Dhaliwal SS, Nahid A-A, Abbas R (2018). Effective intrusion detection system using XGBoost. Information.

[ref-10] Erasmus JJ, Connolly JE, McAdams HP, Roggli VL (2000). Solitary pulmonary nodules: part I. Morphologic evaluation for differentiation of benign and malignant lesions. Radiographics.

[ref-11] Ferlay JEM, Lam F, Colombet M, Mery L, Piñeros M (2020). Global cancer observatory: cancer today.

[ref-12] Fu F, Zhang Y, Wang S, Li Y, Wang Z, Hu H, Chen H (2021a). Computed tomography density is not associated with pathological tumor invasion for pure ground-glass nodules. The Journal of Thoracic and Cardiovascular Surgery.

[ref-13] Fu F, Zhang Y, Wang S, Li Y, Wang Z, Hu H, Chen H (2021b). Computed tomography density is not associated with pathological tumor invasion for pure ground-glass nodules. The Journal of Thoracic and Cardiovascular Surgery.

[ref-14] Godoy MC, Naidich DP (2012). Overview and strategic management of subsolid pulmonary nodules. Journal of Thoracic Imaging.

[ref-15] Goldstraw P, Chansky K, Crowley J, Rami-Porta R, Asamura H, Eberhardt WE, Nicholson AG, Groome P, Mitchell A, Bolejack V (2016). The IASLC lung cancer staging project: proposals for revision of the TNM stage groupings in the forthcoming (eighth) edition of the TNM classification for lung cancer. Journal of Thoracic Oncology.

[ref-16] Gumus M, Kiran MS (2017). Crude oil price forecasting using XGBoost.

[ref-17] Hawkins S, Wang H, Liu Y, Garcia A, Stringfield O, Krewer H, Li Q, Cherezov D, Gatenby RA, Balagurunathan Y (2016). Predicting malignant nodules from screening CT scans. Journal of Thoracic Oncology.

[ref-18] Henschke CI, Yankelevitz DF, Mirtcheva R, McGuinness G, McCauley D, Miettinen OS, Group E (2002). CT screening for lung cancer: frequency and significance of part-solid and nonsolid nodules. The American Journal of Roentgenology.

[ref-19] Huang Y, Liu Z, He L, Chen X, Pan D, Ma Z, Liang C, Tian J, Liang C (2016). Radiomics signature: a potential biomarker for the prediction of disease-free survival in early-stage (I or II) non-small cell lung cancer. Radiology.

[ref-20] Hutchinson BD, Moreira AL, Ko JP (2017). Spectrum of subsolid pulmonary nodules and overdiagnosis. Seminars in Roentgenology.

[ref-21] Jang HJ, Lee KS, Kwon OJ, Rhee CH, Shim YM, Han J (1996). Bronchioloalveolar carcinoma: focal area of ground-glass attenuation at thin-section CT as an early sign. Radiology.

[ref-22] Jiang Y, Che S, Ma S, Liu X, Guo Y, Liu A, Li G, Li Z (2021). Radiomic signature based on CT imaging to distinguish invasive adenocarcinoma from minimally invasive adenocarcinoma in pure ground-glass nodules with pleural contact. Cancer Imaging.

[ref-23] Kitami A, Sano F, Hayashi S, Suzuki K, Uematsu S, Kamio Y, Suzuki T, Kadokura M, Omatsu M, Kunimura T (2016). Correlation between histological invasiveness and the computed tomography value in pure ground-glass nodules. Surgery Today.

[ref-24] Kuriyama K, Seto M, Kasugai T, Higashiyama M, Kido S, Sawai Y, Kodama K, Kuroda C (1999). Ground-glass opacity on thin-section CT: value in differentiating subtypes of adenocarcinoma of the lung. American Journal of Roentgenology.

[ref-25] Lambin P, Leijenaar RT, Deist TM, Peerlings J, De Jong EE, Van Timmeren J, Sanduleanu S, Larue RT, Even AJ, Jochems A (2017). Radiomics: the bridge between medical imaging and personalized medicine. Nature Reviews Clinical Oncology.

[ref-26] Lambin P, Rios-Velazquez E, Leijenaar R, Carvalho S, Van Stiphout RG, Granton P, Zegers CM, Gillies R, Boellard R, Dekker A (2012). Radiomics: extracting more information from medical images using advanced feature analysis. European Journal of Cancer.

[ref-27] Liu S, Wang R, Zhang Y, Li Y, Cheng C, Pan Y, Xiang J, Zhang Y, Chen H, Sun Y (2016). Precise diagnosis of intraoperative frozen section is an effective method to guide resection strategy for peripheral small-sized lung adenocarcinoma. Journal of Clinical Oncology.

[ref-28] McKee BJ, Regis SM, McKee AB, Flacke S, Wald C (2016). Performance of ACR Lung-RADS in a clinical CT lung screening program. Journal of the American College of Radiology.

[ref-29] Meng F, Guo Y, Li M, Lu X, Wang S, Zhang L, Zhang H (2021). Radiomics nomogram: A noninvasive tool for preoperative evaluation of the invasiveness of pulmonary adenocarcinomas manifesting as ground-glass nodules. Translational Oncology.

[ref-30] Nagy-Mignotte H, Guillem P, Vesin A, Toffart AC, Colonna M, Bonneterre V, Brichon PY, Brambilla C, Brambilla E, Lantuejoul S, Timsit JF, Moro-Sibilot D, Multidisciplinary Thoracic Oncology Group at Grenoble University H (2011). Primary lung adenocarcinoma: characteristics by smoking habit and sex. European Respiratory Journal.

[ref-31] Ng Y, Patsios D, Roberts H, Walsham A, Paul N, Chung T, Herman S, Weisbrod G (2008). CT-guided percutaneous fine-needle aspiration biopsy of pulmonary nodules measuring 10 mm or less. Clinical Radiology.

[ref-32] Nioche C, Orlhac F, Boughdad S, Reuzé S, Goya-Outi J, Robert C, Pellot-Barakat C, Soussan M, Frouin F, Buvat I (2018). LIFEx: a freeware for radiomic feature calculation in multimodality imaging to accelerate advances in the characterization of tumor heterogeneity. Cancer Research.

[ref-33] Nomori H, Watanabe K, Ohtsuka T, Naruke T, Suemasu K, Uno K (2004). Evaluation of F-18 fluorodeoxyglucose (FDG) PET scanning for pulmonary nodules less than 3 cm in diameter, with special reference to the CT images. Lung Cancer.

[ref-34] Owens CA, Peterson CB, Tang C, Koay EJ, Yu W, Mackin DS, Li J, Salehpour MR, Fuentes DT, Court LE (2018). Lung tumor segmentation methods: impact on the uncertainty of radiomics features for non-small cell lung cancer. PLOS ONE.

[ref-35] Parmar C, Leijenaar RT, Grossmann P, Rios Velazquez E, Bussink J, Rietveld D, Rietbergen MM, Haibe-Kains B, Lambin P, Aerts HJ (2015). Radiomic feature clusters and prognostic signatures specific for lung and head & neck cancer. Scientific Reports.

[ref-36] Ren X, Guo H, Li S, Wang S, Li J (2017). A novel image classification method with CNN-XGBoost model.

[ref-37] Ricciardi S, Booton R, Petersen RH, Infante M, Scarci M, Veronesi G, Cardillo G (2021). Managing of screening-detected sub-solid nodules—a European perspective. Translational Lung Cancer Research.

[ref-38] Scholten ET, de Jong PA, de Hoop B, van Klaveren R, van Amelsvoort-van de Vorst S, Oudkerk M, Vliegenthart R, de Koning HJ, Van der Aalst CM, Vernhout RM (2015). Towards a close computed tomography monitoring approach for screen detected subsolid pulmonary nodules?. European Respiratory Journal.

[ref-39] She Y, Zhao L, Dai C, Ren Y, Zha J, Xie H, Jiang S, Shi J, Shi S, Shi W (2017). Preoperative nomogram for identifying invasive pulmonary adenocarcinoma in patients with pure ground-glass nodule: a multi-institutional study. Oncotarget.

[ref-40] Shi L, Shi W, Peng X, Zhan Y, Zhou L, Wang Y, Feng M, Zhao J, Shan F, Liu L (2021). Development and validation a nomogram incorporating CT radiomics signatures and radiological features for differentiating invasive adenocarcinoma from adenocarcinoma *in situ* and minimally invasive adenocarcinoma presenting as ground-glass nodules measuring 5–10 mm in diameter. Frontiers in Oncology.

[ref-41] Sun Q, Huang Y, Wang J, Zhao S, Zhang L, Tang W, Wu N (2019). Applying CT texture analysis to determine the prognostic value of subsolid nodules detected during low-dose CT screening. Clinical Radiology.

[ref-42] Sun Y, Li C, Jin L, Gao P, Zhao W, Ma W, Tan M, Wu W, Duan S, Shan Y (2020). Radiomics for lung adenocarcinoma manifesting as pure ground-glass nodules: invasive prediction. European Radiology.

[ref-43] Tang Q, Wu H, Teng G, Bu H, Tan C, Liu J, Zhang X, Zhang Y, Yan W, Deng J (2019). Prediction of casing damage in unconsolidated sandstone reservoirs using machine learning algorithms.

[ref-44] R Core Team (2022).

[ref-45] Torlay L, Perrone-Bertolotti M, Thomas E, Baciu M (2017). Machine learning—XGBoost analysis of language networks to classify patients with epilepsy. Brain Informatics.

[ref-46] Travis WD, Brambilla E, Burke AP, Marx A, Nicholson AG (2015). Introduction to The 2015 world health organization classification of tumors of the lung, pleura, thymus, and heart. Journal of Thoracic Oncology.

[ref-47] Travis WD, Brambilla E, Noguchi M, Nicholson AG, Geisinger KR, Yatabe Y, Beer DG, Powell CA, Riely GJ, Van Schil PE (2011). International association for the study of lung cancer/american thoracic society/european respiratory society international multidisciplinary classification of lung adenocarcinoma. Journal of Thoracic Oncology.

[ref-48] Vickers AJ, Cronin AM, Elkin EB, Gonen M (2008). Extensions to decision curve analysis, a novel method for evaluating diagnostic tests, prediction models and molecular markers.. BMC Medical Informatics and Decision Making.

[ref-49] Wu F, Tian SP, Jin X, Jing R, Yang YQ, Jin M, Zhao SH (2017). CT and histopathologic characteristics of lung adenocarcinoma with pure ground-glass nodules 10 mm or less in diameter. European Radiology.

[ref-50] Yang D, Rao G, Martinez J, Veeraraghavan A, Rao A (2015). Evaluation of tumor-derived MRI-texture features for discrimination of molecular subtypes and prediction of 12-month survival status in glioblastoma. Medical Physics.

[ref-51] Yip R, Henschke CI, Yankelevitz DF, Boffetta P, Smith JP, nternational Early Lung Cancer I (2015). The impact of the regimen of screening on lung cancer cure: a comparison of I-ELCAP and NLST. European Journal of Cancer Prevention.

[ref-52] Ypsilantis P-P, Siddique M, Sohn H-M, Davies A, Cook G, Goh V, Montana G (2015). Predicting response to neoadjuvant chemotherapy with PET imaging using convolutional neural networks. PLOS ONE.

[ref-53] Zhang T, Pu XH, Yuan M, Zhong Y, Li H, Wu JF, Yu TF (2019). Histogram analysis combined with morphological characteristics to discriminate adenocarcinoma *in situ* or minimally invasive adenocarcinoma from invasive adenocarcinoma appearing as pure ground-glass nodule. European Journal of Radiology.

[ref-54] Zhang Y, Jheon S, Li H, Zhang H, Xie Y, Qian B, Lin K, Wang S, Fu C, Hu H, Zheng Y, Li Y, Chen H (2020). Results of low-dose computed tomography as a regular health examination among Chinese hospital employees. The Journal of Thoracic and Cardiovascular Surgery.

[ref-55] Zhao W, Xu Y, Yang Z, Sun Y, Li C, Jin L, Gao P, He W, Wang P, Shi H, Hua Y, Li M (2019). Development and validation of a radiomics nomogram for identifying invasiveness of pulmonary adenocarcinomas appearing as subcentimeter ground-glass opacity nodules. European Journal of Radiology.

