# Supplementary material for: Computed tomography-based radiomics machine learning models for prediction of histological invasiveness with sub-centimeter subsolid pulmonary nodules: a retrospective study
Source: PeerJ. 2023 Jan 10;11:e14559. doi: 10.7717/peerj.14559 (PMC9838201; doi:10.7717/peerj.14559)
Supplement: Supplemental Information 3 [file peerj-11-14559-s003.docx]

SEX: 1 = male, 2 = female

LOCATION: 1 = Left Upper Lobe, 2 = Left Lower Lobe, 3 = Right Upper Lobe, 4 = Right Upper Lobe, 5 = Right Lower Lobe

LABEL: 1 = Group (IAC), 0 = Group (AAH/AIS/MIA)

LOBULATION: 1 = Present, 0 = Absent

VASCULAR BREAKTHROU-GH SIGN: 1 = Present, 0 = Absent

BUBBLE-LIKE LUCENCY: 1 = Present, 0 = Absent

MARGIN: 1 = Unclear, 0 = Clear

SHARP: 1 = Irregular or polygonal, 0 = Round or oval

SPICULATION: 1 = Present, 0 = Absent

PLERUAL INDENTATION SIGN: 1 = Present, 0 = Absent

CAVITY: 1 = Present, 0 = Absent
